# Research on construction deformation prediction and disaster warning of karst slope based on mutation theory

**DOI:** 10.1038/s41598-022-19380-5

**Published:** 2022-09-07

**Authors:** Yanli Qi, Gang Tian, Mingzhou Bai, Linlin Song

**Affiliations:** 1 School of Civil Engineering, Beijing Jiaotong University, Beijing, 100044 China; 2 China Electronic Engineering Design Institute Co., Ltd., Beijing, 100142 China; 3 C+E Center for Engineering Research Test and Appraisal Co., Ltd., Beijing, 100142 China

**Keywords:** Environmental sciences, Environmental social sciences, Natural hazards, Solid Earth sciences

## Abstract

In the study of deformation prediction and disaster warning during karst slope construction, the influencing factors and deformation law should be comprehensively considered. The layout of the deformation monitoring points of karst slope is affected by the thickness of karst overburden soil, dissolution and fragmentation degree, karst development degree, slope cracking degree, fault or weak interlayer and other factors. In this paper, the author aimed at the problem of construction deformation prediction and disaster warning of karst slope, proposed an improved model of cusp mutation by applying and optimizing the cusp mutation model, analysed the deformation trend and sudden change type of the slope, and obtained the critical control early warning value of slope deformation. Therefore, it is feasible to analyse the deformation and mutation characteristics of karstified slope by using a virtual reality-mutation model. In addition, based on the empirical formula of the slope sliding limit deformation rate and grey prediction model, the critical control warning value of slope deformation is obtained, which provides a basis to quantify the deformation index of risk evaluation. This method provides a new idea to predict karst slope construction deformation and catastrophic deformation warning and has a reference value for similar engineering examples.

## Introduction

Karst is a general term for the geological processes where water acts on soluble rocks (carbonate rocks, gypsum, rock salt, etc.) mainly via chemical dissolution. This phenomenon is supplemented by mechanical processes such as erosion, latent erosion and collapse of flowing water, and the phenomena produced by these processes. In recent years, construction deformation of karst slope has a concern. On one hand, during the construction period of the cutting slope, the construction disturbance destroys the original balance state of the mountain, makes the slope produce excessive deformation and even induces the slope to become unstable. On the other hand, the mountains in karst areas have been corroded for a long time, and the internal structure is mostly damaged, which results in low strength and poor stability of the rock mass structure. This makes the deformation prediction of karst slope during construction more complicated and catastrophic deformation warnings more difficult. At this time, the deformation of karst slope during construction is particularly important, especially the deformation prediction and catastrophic deformation warning of karst slope during construction.

To comprehensively study the deformation prediction and catastrophic deformation warning of karst slope during construction, scholars at home and abroad have performed many studies^1–6^ to promote theoretical development and provide technical guidance for engineering. Studies on this issue are mainly divided into three categories: karst slope catastrophe mechanism^7–15^, karst slope stability evaluation^16–20^, and early warning research based on slope monitoring data^21–23^.

Research on karst slope disaster mechanism mainly focuses on the displacement change rule, deformation failure mode, deformation failure mechanism, instability mechanism, etc. Xu et al.^7^ analysed the deformation and failure mechanism of three typical types of mixed soil–rock slopes, revealed the typical disaster modes, and proposed disaster control measures in the entire process. Liu et al.^8^ used the geological historical process mechanism analysis method and numerical simulation method to study the deformation and failure mechanism of a layered gently inclined hornstone slope in the karst area of Guizhou, and four types of deformation and failure mechanisms were summarized. Xiong et al.^9^ studied the engineering geological conditions of the steep cliff of Shenxiu Cave and used FLAC3D for numerical simulation to study the layered rock foundation pit slope. Aiming at a high fill project at the southern end of Chongqing Wulong Civil Airport, Xiong et al.^10^ analysed the displacement distribution characteristics and deformation development law of fillers, dam bodies and dissolved chambers and selected the optimal karst treatment scheme by comparison. Shi^11^ systematically summarized the karst distribution law, development characteristics and instability mechanism of the roadbed section of the Shanghai-Kunming High-speed Railway (Guizhou section). Liang Tang took the drink horse pond landslide as the main research object and analysed its deformation failure mode. The use of 3DEC simulation reproduced the landslide deformation and failure and movement process^12^. Based on the research and data collection, Hua Huang thoroughly studied the North Slope of the Xianfeng opencast coal mine deformation destruction mechanisms and stability evaluation^13^. Baiping Song concluded with geological properties, potential dangers, hazard causes and analysed the geological inducing and damages in geologically complicated Yunnan. Meanwhile, the inducing mechanism and inducing chains for highway engineering geological environment are provided in the Paper^14^. He^15^ took the Wangxia unstable rocks on the WU Gorge bank slope as an example and studied the formation and failure mechanism, lasting effect and hazard assessment.

Research on karst slope stability evaluation mainly focuses on influencing factors, stability analysis, stability evaluation, dynamic stability, etc. Liu et al.^16^ took the Changsha Ice & Snow World mine project as an example to study the dynamic stability of steep rocky high slope by combining on-site measurement and indoor numerical analysis. Based on the background of a building foundation pit slope engineering project in Chenzhou city, Dajian Li established the model of experimental analysis based on the two typical karst cave sections in the slope. Through model tests and numerical simulations, the deformation and stability characteristics of building slope containing karst caves have been studied^17^. Based on the background of karst development in Guangxi, Zhao^18^ analysed the karst problems that affected the stability of foundation pit slope. Based on the concrete project, Chen^19^ analysed the stability of the high reinforcement slope, pile anchor slope and karst foundation using theoretical, FlAC3D numerical and field monitoring.

Early warning research based on slope monitoring data^21–23^ mainly focuses on numerical simulation calculations, prediction models, the selection of calculation parameters, etc. Gao et al.^21^ addressed the problem of the fuzzy selection basis of dual-structure slope simulation calculation parameters and the complicated force and deformation of the slope body in the supporting process. Gao et al.^22^ studied the problem of the loss of anchor cable prestress over time in a soil–rock dual-structure slope. Gao et al.^23^ proposed and used the deep displacement monitoring data and the p value test method to check the simulation parameters.

The above studies involve the catastrophe mechanism of karst slopes and stability evaluation of karst slopes, but relatively few studies have both. In addition, most of the above studies involve monitoring data processing, model testing and numerical simulation, but there is less theoretical research.

Based on the application and optimization improvement of mutation theory and the cusp mutation model in slope deformation, the virtual reality grey trend-cusp mutation model (short for virtual reality-mutation model) is proposed in this paper. This model is applied to the Damao slope, and the study shows that the mutation effect can be transformed between two deformation trends or states, including convergence–divergency mutation type (C–D), divergence–convergence mutation type (D–C), convergence–convergence mutation type (C–C), and divergence–divergency mutation type (D–D). Thus, the main deformation and mutation forms of the Damao slope are the C–D mutation type, D–C mutation type and C–C mutation type. Among them, the C–D mutation type greatly affects the deformation state of the Damao slope and easily causes its cracking and failure. Therefore, it is feasible to analyse the deformation and mutation characteristics of karstified slope using the virtual reality-mutation model. In addition, based on the empirical formula of the slope sliding limit deformation rate and grey prediction model, the critical control warning value of slope deformation is obtained, which provides a basis to quantify the deformation index of risk evaluation. Theoretical analysis, field data processing, and early warning research based on slope monitoring data^21–23^, prove that the proposed virtual reality-mutation model in this paper and the critical control warning value of catastrophic deformation are scientific, reasonable and effective. This method provides a new idea to predict karst slope construction deformation and catastrophic deformation warning and has a certain reference value for similar engineering examples.

## Material

### Overview of the study area

**Figure 1:**
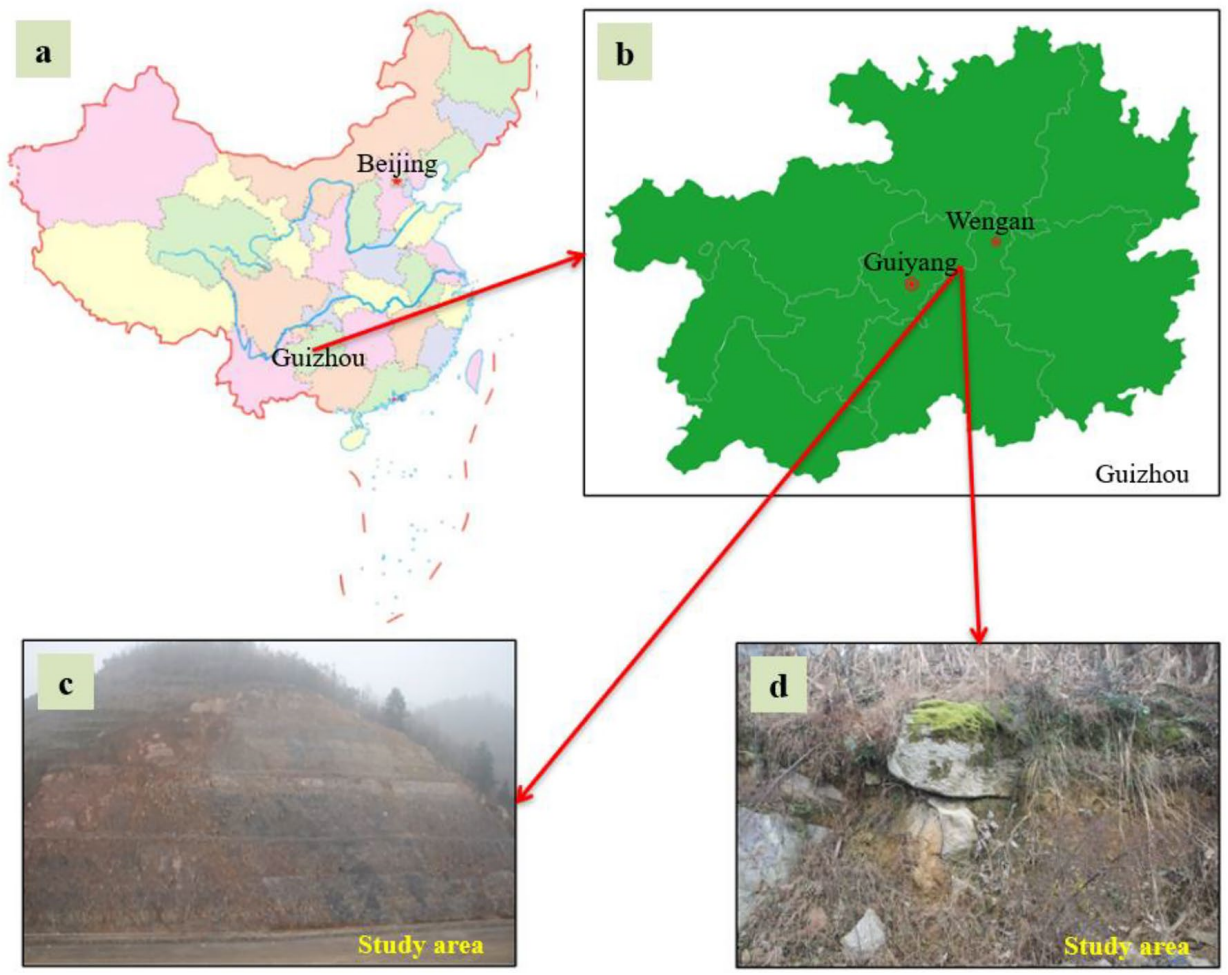
Site condition of landslide. (**a**) Map of China. The base map is accessible on https://cn.bing.com/images/search?view=detailV2&ccid=MLo786tp&id=4F452A89865BCBE9C761A4046E70743661C7DF99&thid=OIP.MLo786tp5RkhIDkBbRGnVwHaGE&mediaurl=https%3A%2F%2Fts1.cn.mm.bing.net%2Fth%2Fid%2FR-C.30ba3bf3ab69e519212039016d11a757%3Frik%3Dmd%252fHYTZ0cG4EpA%26riu%3Dhttp%253a%252f%252fxysj.cditv.cn%252f2019%252f0801%252f20190801121423480.jpg%26ehk%3D3JHW%252fsLoy5mdrFyA0wJJ7Gabtv5QCgbJ7HaPjhZ84dQ%253d%26risl%3D%26pid%3DImgRaw%26r%3D0%26sres%3D1%26sresct%3D1&exph=495&expw=604&q=%e4%b8%ad%e5%9b%bd%e5%9c%b0%e5%9b%be&simid=607993912997916131&form=IRPRST&ck=E673B0A0162B076A9FA86AD609AA35DD&selectedindex=100&ajaxhist=0&ajaxserp=0&vt=0&sim=11 (August 2022). (**b**) Map of Guizhou Province. The base map is accessible on https://cn.bing.com/images/search?view=detailV2&ccid=U7NXMWND&id=0AA693DA99B737B85748BDFA532DD2898F136C31&thid=OIP.U7NXMWNDa5iEh7mUObbEQgHaFp&mediaurl=https%3a%2f%2fts1.cn.mm.bing.net%2fth%2fid%2fR-C.53b3573163436b988487b99439b6c442%3frik%3dMWwTj4nSLVP6vQ%26riu%3dhttp%253a%252f%252fwww.weather.com.cn%252fm2%252fi%252fguizhou%252fjtlycp%252fgzdt.png%26ehk%3dbxLizw0m%252f9iEAy29W7pcAhv%252b2u8K6IeOu1V5LFuALBA%253d%26risl%3d%26pid%3dImgRaw%26r%3d0&exph=763&expw=1000&q=%e8%b4%b5%e5%b7%9e%e5%9c%b0%e5%9b%be&simid=608052217180004736&FORM=IRPRST&ck=52C4AB36667277872C78A38DF239915D&selectedIndex=47&ajaxhist=0&ajaxserp=0 (August 2022). (**c**) Study area picture. (d) The site condition of slope. The images of c and d were taken by Gang Tian (one of your co-authors).

The Damao slope is located in guiweng (Guiyang to Weng'an) highway in central Guizhou, which belongs to the middle and low mountain karst landform, with limestone as the main formation lithology and 310°∠16° occurrence. The original mountain natural slope is 13°–32° with large fluctuations. The surface of the mountain is mostly covered by a karstic overburden soil layer with uneven thickness. Vegetation grows at the top of the mountain, and there are small terraced fields on both sides of the mountain. A small amount of rock mass is exposed on the local surface of the mountain, and most of them show corrosion characteristics, as shown in Fig. 1.

### Deformation monitoring data during construction

#### Deformation monitoring point layout theory

The layout of the deformation monitoring points of karst slope is affected by the thickness of karst overburden soil, dissolution and fragmentation degree, karst development degree, slope cracking degree, fault or weak interlayer and other factors. In the study of deformation prediction and disaster warning during karst slope construction, the influencing factors and deformation law should be comprehensively considered.

The selection and arrangement of monitoring points for karst slope should be representative and can accurately reflect the deformation of karst slope. The author determines the monitoring point location according to the engineering analogy method and expert experience method. Moreover, in areas with strong karst development and poor geological and hydrological conditions, monitoring points should be arranged based on the optimal placement method of slope monitoring points based on the principle of error analysis.

Stable state F of a karst slope is mainly affected by the internal gestate disaster factor (IGDF) and external gestate disaster factor (EGDF). Its function expression is,

1 $$F=g\left(I,E\right)$$

The karst slope is divided into m (m → ∞) sections for stable state analysis, and the sections with the most serious influence of internal and external gestate disaster factors (I,E) = Max [(I1,E1), (I2,E2)…, (Im,Em)] are selected to determine the slope state. When the stable state of the karstified slope is Fi, the corresponding internal and external gestate disaster factors of the karst slope are (Ii,Ei), but it is actually (Ii + ΔI,Ei + ΔE).

According to Taylor's formula, Eq. (1) is expanded at (Ii,Ei), the first term is taken, and the state change of the karst slope caused by the judgement deviation of internal and external gestate disaster factors is as follows:

2 $$\Delta F\approx {g}_{I}\left({I}_{i},{E}_{i}\right)\Delta I+{g}_{E}\left({I}_{i},{E}_{i}\right)\Delta E$$

Combining Equation $\Delta F = F^{\prime} - F_{i} \approx f^{\prime}\left( {x_{i} } \right)\Delta x$ with Eq. (2), we obtain

3 $${f}^{^{\prime}}\left({x}_{i}\right)\Delta x={g}_{I}\left({I}_{i},{E}_{i}\right)\Delta I+{g}_{E}\left({I}_{i},{E}_{i}\right)\Delta E$$

We assign a value, $\Delta {F}_{i}={g}_{i}\left({I}_{i},{E}_{i}\right)\Delta I$, $\Delta {F}_{E}={g}_{E}\left({I}_{i},{E}_{i}\right)\Delta E$, then,

4 $${f}_{\left({x}_{i}\right)}^{^{\prime}}\Delta x=\Delta {F}_{I}+\Delta {F}_{E}={g}_{I}\left({I}_{i},{E}_{i}\right)\Delta I+{g}_{E}\left({I}_{i},{E}_{i}\right)\Delta E$$

where ΔI and ΔE are the difference between actual value and judgement value of internal and external disasters gestate factors respectively; ΔF_I_ and ΔF_E_ are the difference in slope state caused by the judgement deviation of internal and external disaster gestate factors respectively.

When the slope is in a certain state, its external disaster gestate factors are easy to determine, such as load, rainfall and temperature; i.e., the deviation of E_i_ from E_i_ + ΔE is small, ΔE tends to 0, and ΔF_E_ tends to 0. However, the internal disaster gestate factors are concealed in the interior of the slope, such as lithologic characteristics, geological and hydrological conditions, and karst development, and are difficult to determine, and the spatial difference is large, which results in the serious deviation of I_i_ from I_i_ + ΔI; i.e., ΔI is large. Therefore, Eq. (3) can be rewritten as

5 $${f}^{^{\prime}}\left({x}_{i}\right)\Delta x={g}_{I}\left({I}_{i},{E}_{i}\right)\Delta I$$

According to Eq. (5), Δx is directly proportional to Δ*I* when ${f}^{^{\prime}}\left({x}_{i}\right)$ and ${g}_{I}\left({I}_{i},{E}_{i}\right)$ do not change. In other words, in a certain spatial area of karstified slope, a greater impact of the internal disaster gestate factors (I) on the slope corresponds to a greater deformation monitoring value (X) of this area and a more reasonable stable state (F) of the corresponding karstified slope in this area.

Therefore, considering the characteristics of the disaster pregnant factors in the karst slope, the monitoring points should be arranged in areas with strong karst development, poor geological and hydrological conditions (i.e., the karst overburden soil is thicker), dissolution, fragmentation, cave development, slope cracking, fault and weak interlayer.

### Deformation monitoring scheme during the construction period

**Figure 2:**
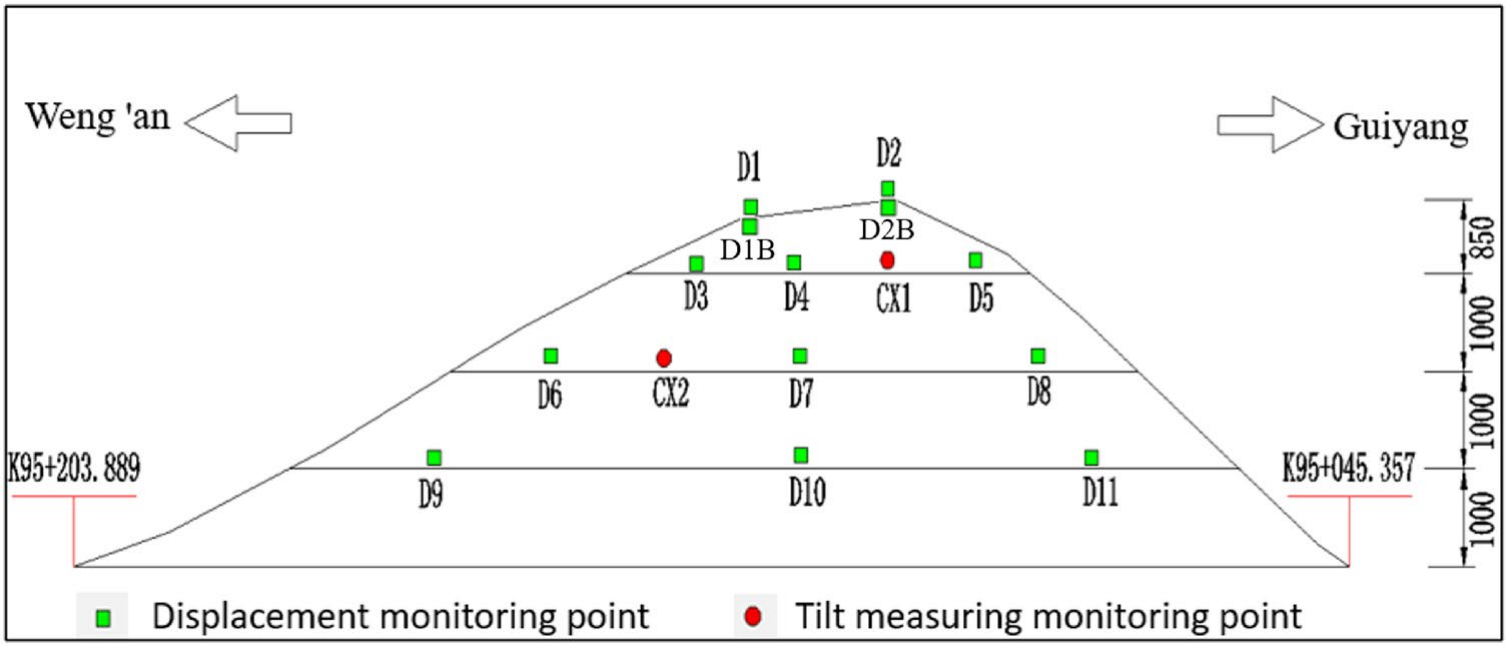
Deformation monitoring point layout of the Damao slope.

According to the layout theory of monitoring points, the deformation monitoring points of the Damao slope were arranged in the form of "dense at the top and sparse at the bottom", as shown in Fig. 2. Because the Damao slope is a bedding slope with a slow dip angle, the monitoring items mainly include slope surface displacement and deep horizontal displacement.

In addition, to avoid mutual interference between slope monitoring and construction, monitoring points were arranged step by step with the progress of slope construction, as shown in Table 1.

**Table 1:** Monitoring point layout time.

Monitoring project	Location	Serial number	Start time of monitoring	Slope excavation
Slope surface displacement	Level 4 slope	D1	2014.04.26	Excavated to the middle and lower part of the fourth stage
		D2	2014.04.26	
		D1B	2014.07.26	Excavated to the middle and lower part of the first stage
		D2B	2014.07.26	
	Level 3 slope	D3	2014.05.24	Excavated to the middle and lower part of the third stage
		D4	2014.05.24	
		D5	2014.05.24	
	Level 2 slope	D6	2014.06.21	Excavated to the middle and lower part of the second stage
		D7	2014.06.21	
		D8	2014.06.21	
	Level 1 slope	D9	2014.07.26	Excavated to the middle and lower part of the first stage
		D10	2014.07.26	
		D11	2014.07.26	
Deep horizontal displacement	Level 3 slope	CX1	2014.07.26	
	Level 2 slope	CX2	2014.07.26	

### Analysis of deformation data

**Figure 3:**
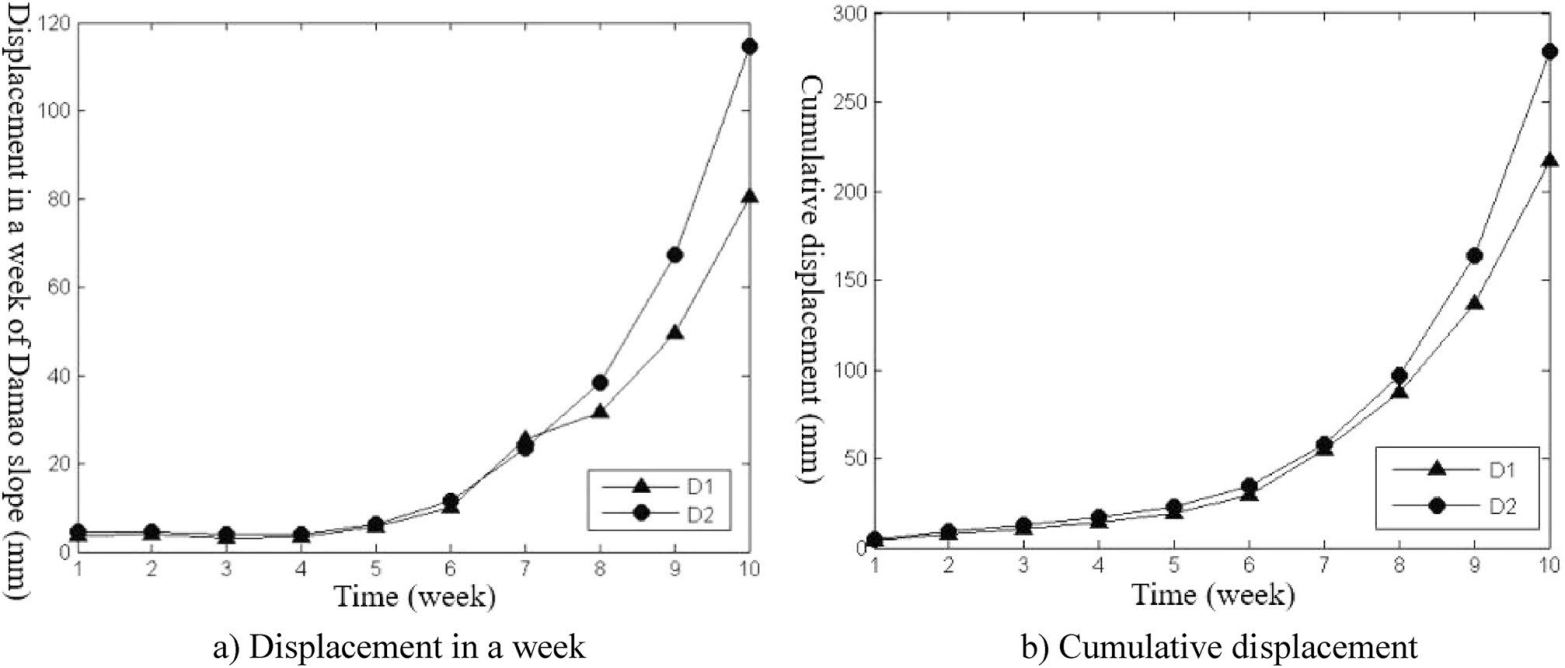
Displacement curves of monitoring points on the slope top from April 26 to June 28.

**Figure 4:**
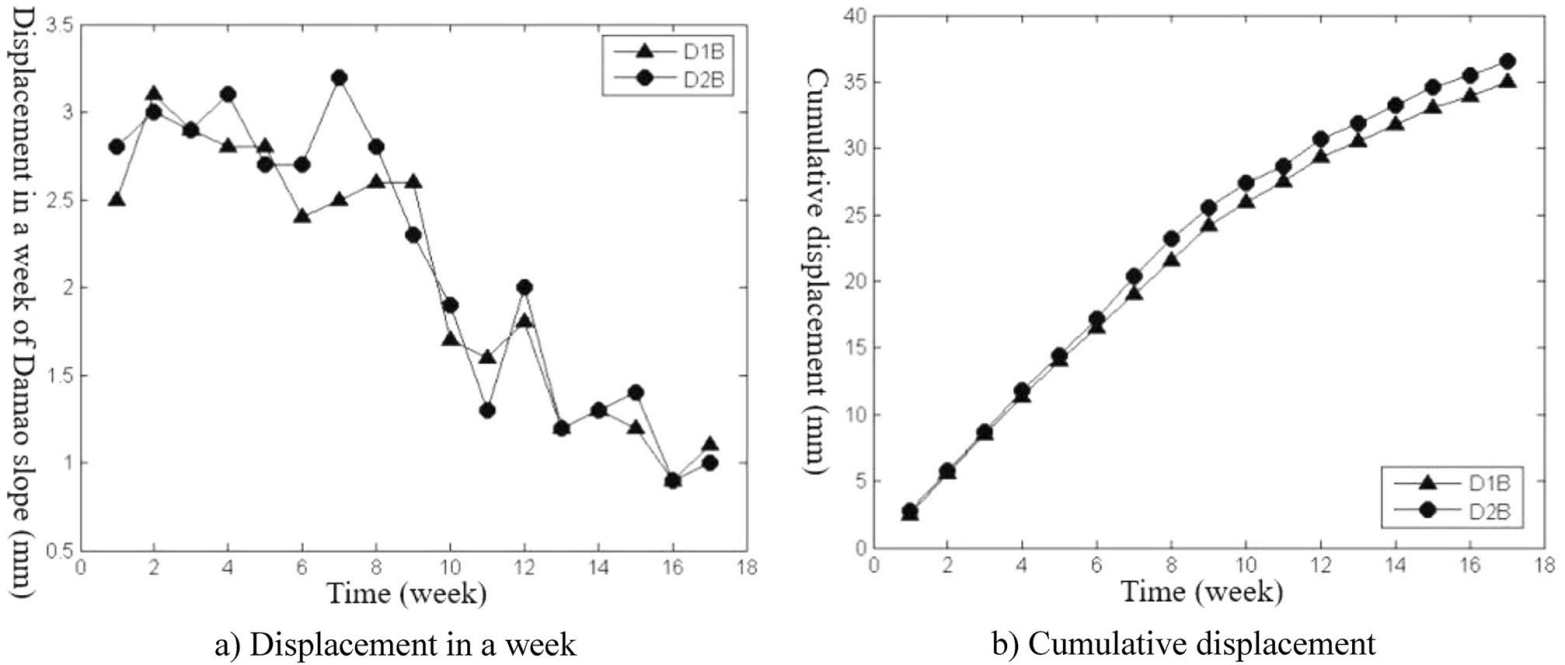
Displacement curves of monitoring points on the slope top from July 26 to November 15.

**Figure 5:**
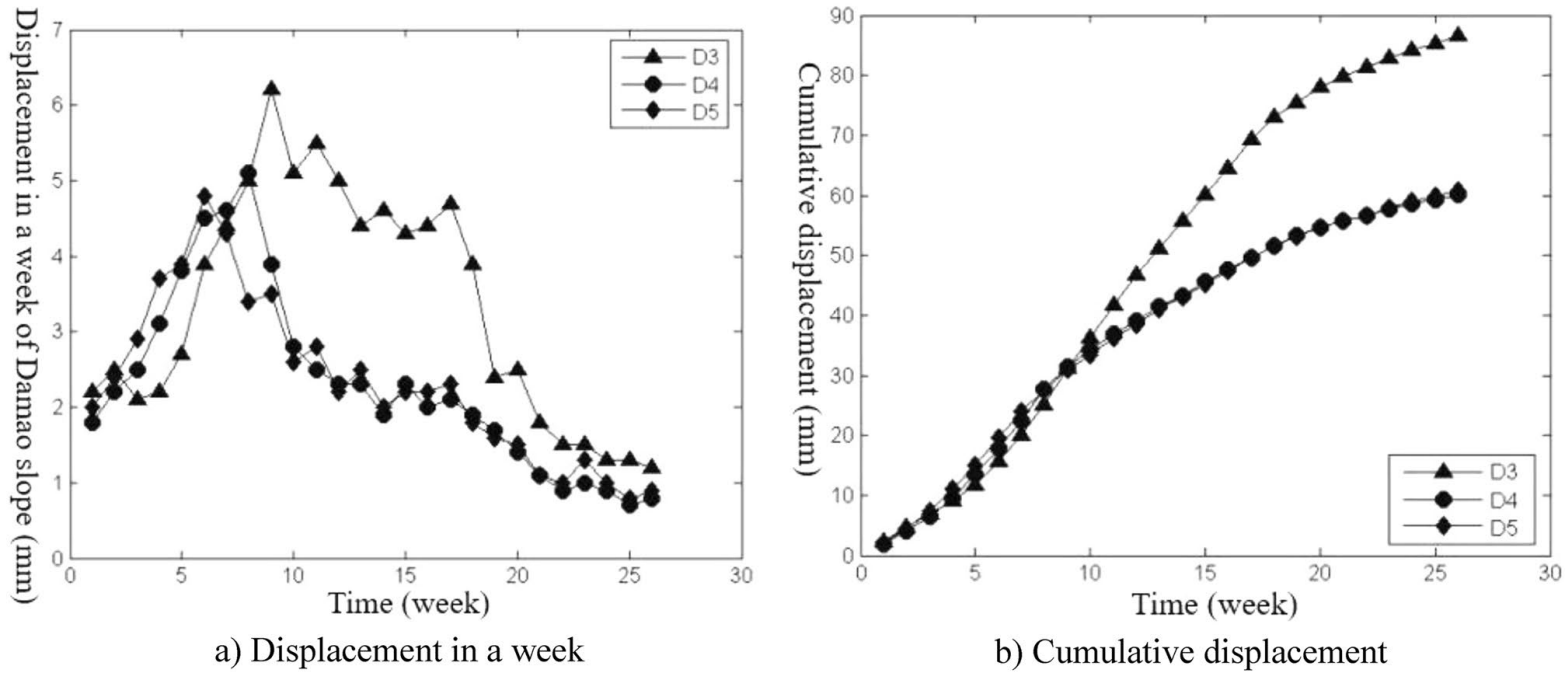
Displacement curves of third-level platform monitoring points from May 24 to November 15.

**Figure 6:**
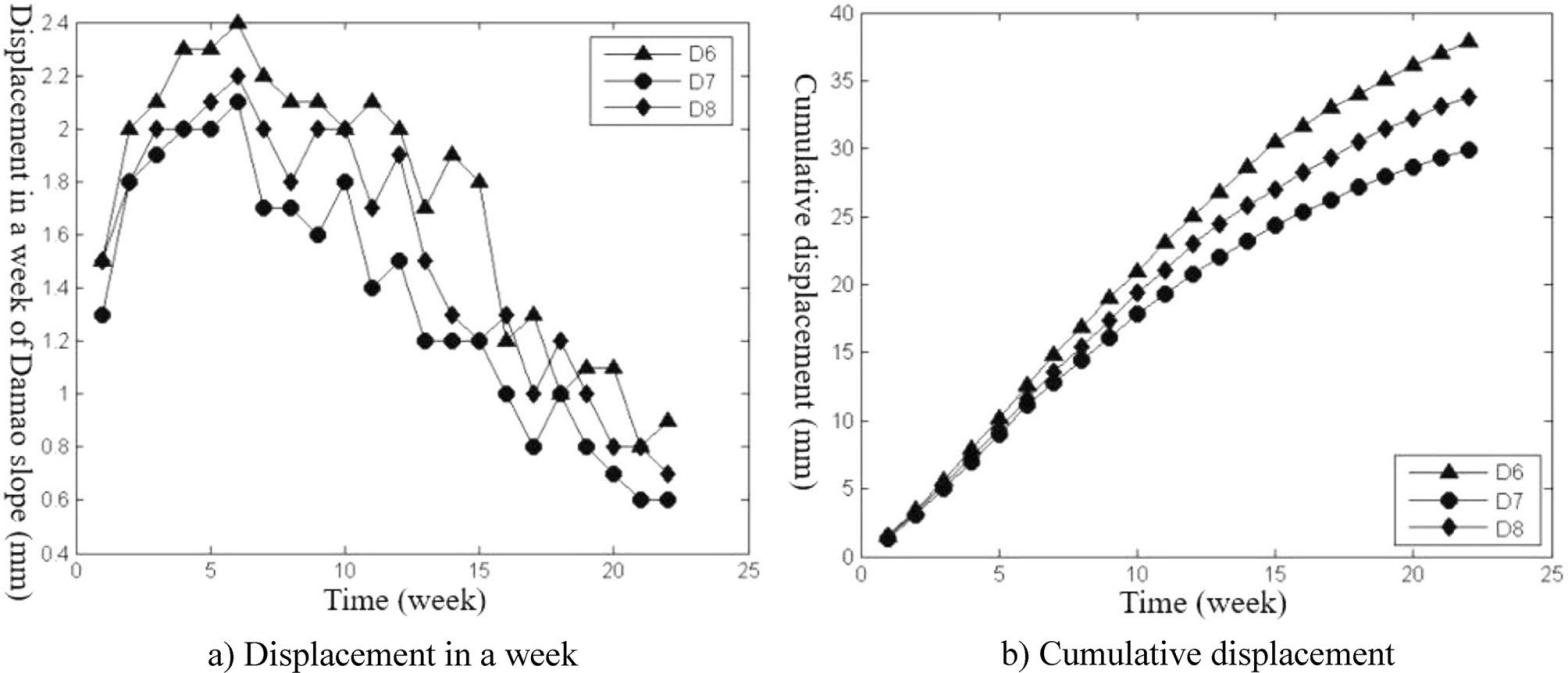
Displacement curves of the second-level platform monitoring points from June 21 to November 15.

**Figure 7:**
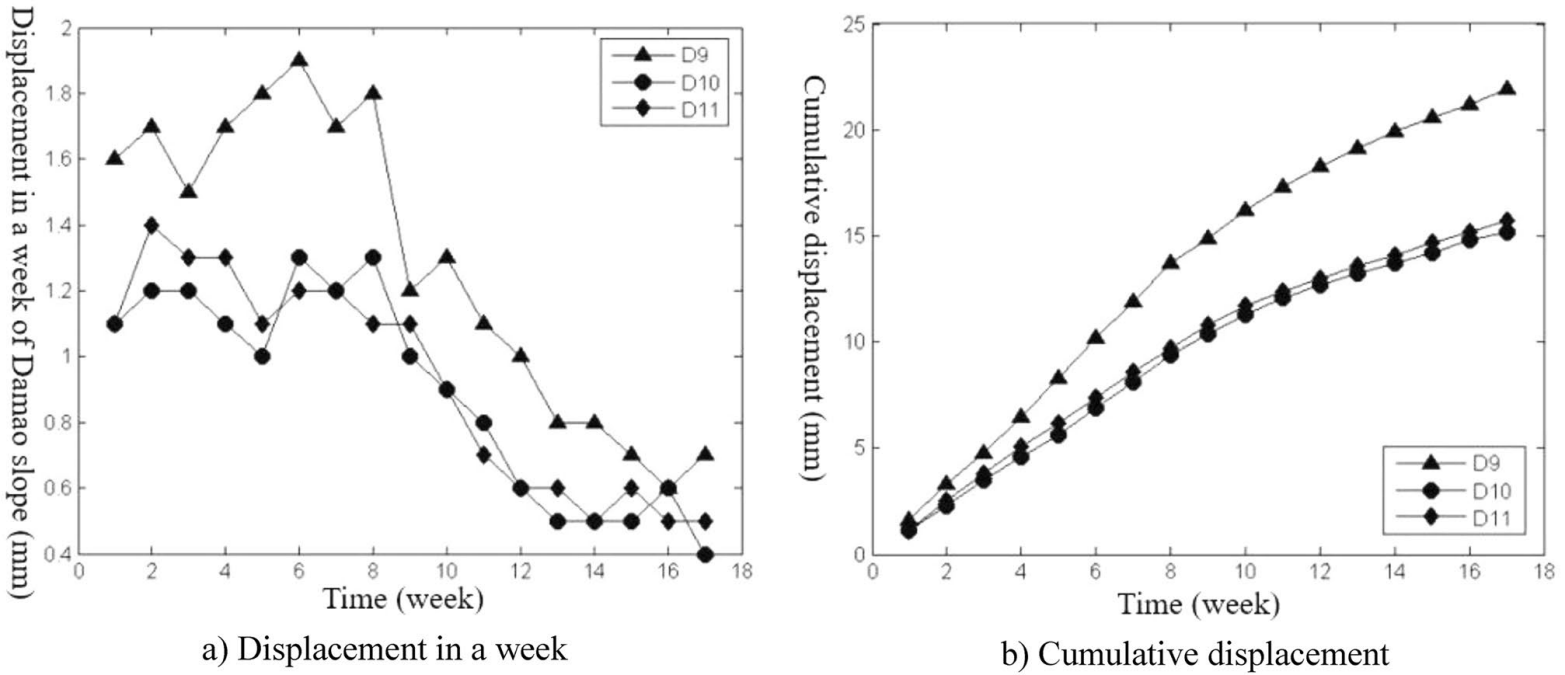
Displacement curves of the first-level platform monitoring points from July 26 to November 15.

**Figure 8:**
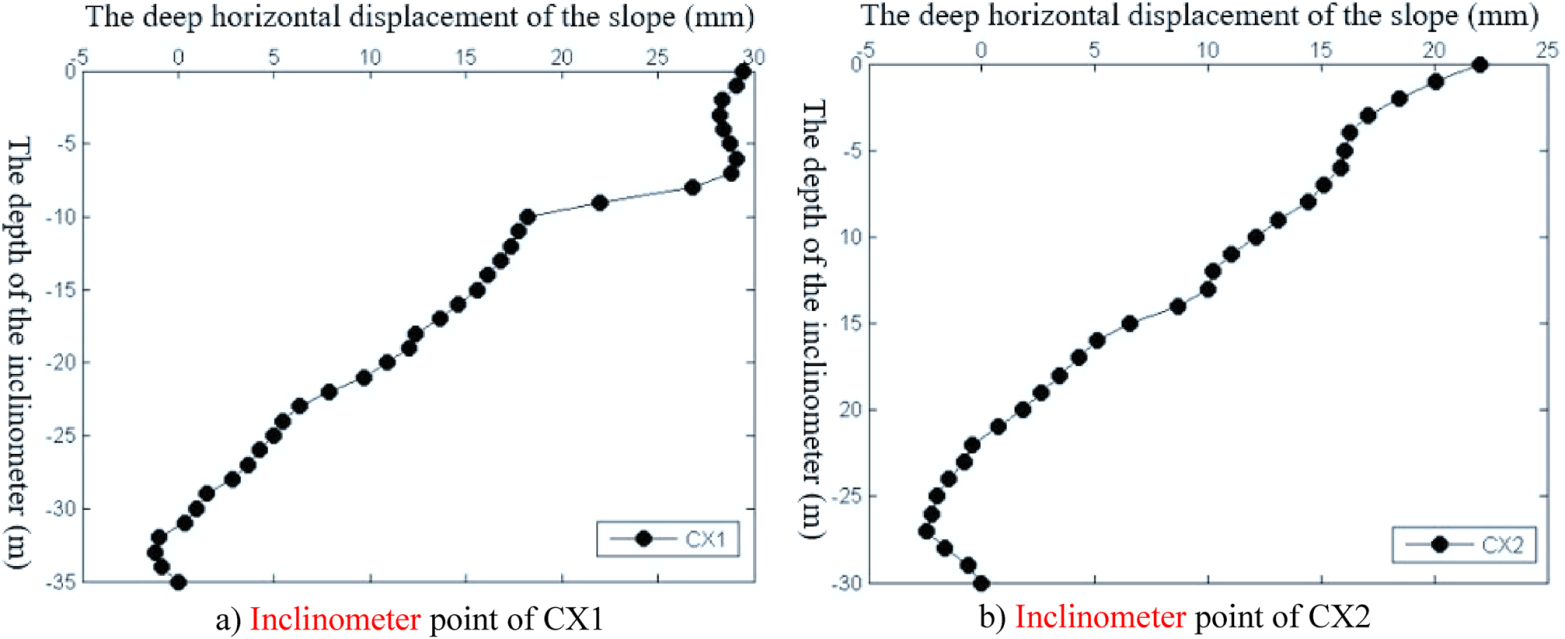
Accumulated deformation curve of the inclinometer point of the Damao slope from July 26 to November 15.

The slope construction monitoring data from April 26, 2014, to November 15, 2014, were selected for analysis, and the slope deformation data were transformed into equal time interval series with “week” as the unit, as shown in Figs. 3, 4, 5, 6, 7, and 8. The cumulative deformation and on-site disaster of the Damao slope are shown in Table 2.

**Table 2:** Cumulative deformation and on-site disaster of Damao slope.

Monitoring location	Starting time	Height (m)	No	Accumulated deformation (mm)	On-site disaster
The level 4 slope	4.26–6.28	30–38	D1	217.0	Slope cracking and instability trend
			D2	278.4	
	7.26–11.15	30–36	D1B	35.0	Steady after cutting the slope
			D2B	36.5	
The level 3 slope	5.24–11.15	20–30	D3	86.6	Partial cracking of slope body
			D4	60.1	The deformation was large in the early stage and stabilized in the late stage
			D5	60.7	
The level 2 slope	6.21–11.15	10–20	D6	37.9	The slope has no obvious abnormality
			D7	29.9	
			D8	33.8	
The level 1 slope	7.26–11.15	0–10	D9	21.9	
			D10	15.2	
			D11	15.7	

Figures 3, 4, 5, 6, 7 and 8, Tables 2 and 3 show that there are deformation differences, i.e., spatial differences and temporal differences, at each monitoring point of the Damao slope.

**Table 3:** Comparison of the deformation correlation degree at each monitoring point.

Points that are associated	Grey absolute correlation	Grey relative correlation	Grey comprehensive correlation
D1&D2	0.8995	0.9971	0.9483
D1B&D2B	0.8411	0.9677	0.9044
D3&D4	0.7549	0.9432	0.8491
D3&D5	0.6738	0.9032	0.7885
D4&D5	0.8409	0.9548	0.8979
D6&D7	0.6886	0.9569	0.8228
D6&D8	0.6491	0.9476	0.7984
D7&D8	0.8953	0.9972	0.9463
D9&D10	0.8411	0.9941	0.9176
D9&D11	0.7991	0.9802	0.8897
D10&D11	0.9384	0.9859	0.9622

1. Spatial difference characteristics
  1. In the direction of height, the cumulative deformation of the Damao slope gradually decreased from top to bottom. Among them, the level-3 slope and level-4 slope had more obvious deformation. On June 28, 2014, the cumulative deformation of the level-4 slope was 217.0–278.4 mm. On November 15, 2014, the accumulated deformation of the level-3 slope was 60.1–86.6 mm, and the tilt measurement data showed that there might be a sliding zone at 6–9 m of the lower part of the third-grade slope. However, by November 15, 2014, the cumulative deformation of the level-1 slope and level-2 slope was only 15.2–21.9 mm and 29.9–37.9 mm, respectively. The tilt measurement data also showed that the level-1 slope and level-2 slope had no obvious sliding zone and were relatively stable overall.
  2. In the horizontal direction, Table 3 shows that the deformation correlation degrees of monitoring points at all levels of slope body were calculated, except for monitoring points of the level-4 slope (only two monitoring points). The deformation correlation degree between the monitoring points on the left side and the monitoring points on the middle and right sides of the level-1, -2, and -3 slopes of the Damao slope is 0.6385–0.8411, and the deformation correlation degree between the right monitoring point and the middle monitoring point is 0.8409–0.9972. Thus, the deformation correlation of the middle and left slopes is lower than that of the middle and right slopes; i.e., the middle and right slopes have better integrity. By analysing the accumulated deformation of slope monitoring points at the same level, we obtain that the accumulated deformation on the left side of the Damao slope is greater than that in the middle and at the right monitoring points. This result shows that the left slope has more severe deformation than the middle and right slopes. Therefore, it is necessary to strengthen the monitoring work on the left side of the Damao slope.
2. Time difference characteristics
  Figures 3, 4, 5, 6, 7 and 8 show that the period of severe deformation of the Damao slope occurs from April to September, i.e., the construction stage of the Damao slope and the period of frequent regional rainfall. After September, the slope shows convergence characteristics, and the slope deformation tends to be stable as a whole. To eliminate the adverse effects of level-4 slope cracking and sliding and level-3 slope deformation accelerating growth, in the middle and early stages of Damao slope construction, from July 3, 2014, to July 13, 2014, a slope cutting treatment and local reinforcement were performed on the top of the Damao slope. Therefore, in general, the time difference characteristics of the deformation of the Damao slope are obviously affected by external factors.

## Methods

### Analysis model of slope deformation characteristics based on mutation theory

#### Mutation theory

Mutation theory is a mathematical theory that solves discontinuity problems based on mutation and leap phenomena. Mutation theory is the theory of singularities. Using topology as a tool and based on the theory of structural stability, the mutation model of related systems is established, as shown in Table 4. It is used to study the problem of “unstable singularities” of the model, to determine and predict whether the continuous gradual change of some control variables will abruptly change the system state during system evolution, i.e., whether the system will change or jump from one state form to another state form, such as sudden destruction of structure, sudden cracking of rock and soil mass, sudden instability collapse of slope engineering, etc. All of these problems can be modelled and analysed using mutation theory.

**Table 4:** Several common mutation models.

Mutation type	Number of control variables	Number of state variables	Potential function
Folding type	1	1	*x*^3^ + *ux*
Cusp type	2	1	*x*^4^ + *ux*^2^ + *vx*
Dovetail type	3	1	*x*^5^ + *ux*^3^ + *vx*^2^ + *wx*
Butterfly type	4	1	*x*^5^ + *tx*^4^ + *ux*^3^ + *vx*^2^ + *wx*
Hyperbolic umbilical type	3	2	*x*^3^ + *y*^3^ + *wxy* − *ux* − *vx*
Elliptic umbilical type	3	2	1/3*x*^3^ − *xy*^2^ + *w*(*x*^2^ + *y*^2^) − *ux* + *vy*
Parabolic umbilical type	4	2	*y*^4^ + *x*^2^y + *wx*^2^ + *ty*^2^ − *ux* − *vy*

### Establishment of the cusp mutation model for slope deformation

In engineering applications, three types of abrupt change models are commonly used: the cusp type, folding type and dovetail type, among which the cusp model is the most widely used because it can easily construct a critical surface and has an intuitive geometry. The cusp mutation model has five typical characteristics^24–26^:

1. Dual mode. When the system is under the action of the same control parameter, two different states may appear, i.e., jump from one mode to another mode.
2. Unreachable. It is impossible for the system to achieve stable equilibrium in the real sense on some state variables, i.e., the system has unstable equilibrium positions, which are not differentiable and transient.
3. Abrupt. A small change in control parameters will cause a huge change in state variables, which will make the system jump from one critical point of local minimum to another critical point of local minimum.
4. Divergence. For continuous smooth changes, small perturbations of control parameters only cause small increments of state variables. However, in the neighbourhood of the degradation point, a small change in parameters will cause a large change in state variables.
5. Hysteresis. Hysteresis occurs when a physical system cannot repeat a process of change in a strict reversed-phase.

**Figure. 9:**
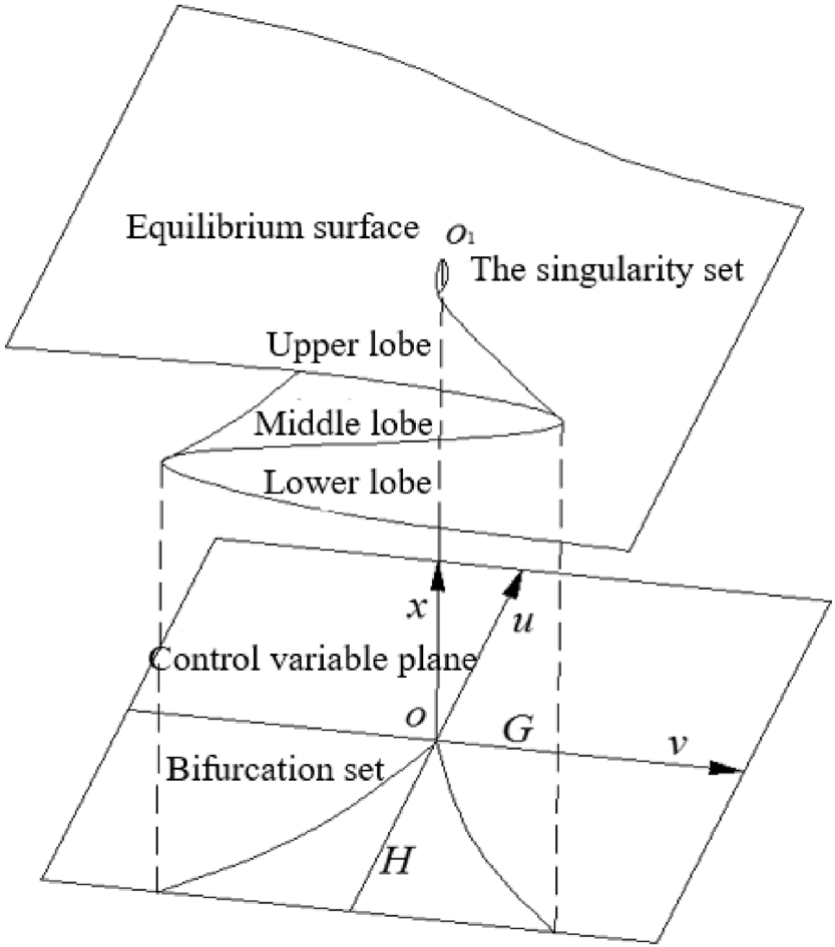
Equilibrium surface and bifurcation set of the cusp mutation model.

To apply the cusp mutation model, the general form of the potential function in the cusp mutation model should be constructed first^27–29^, i.e.,

6 $$ V\left( x \right) = x^{4} + ux^{2} + vx $$

where x is the state variable, and u and V are control variables. Then, the phase space of Eq. (6) is in three-dimensional form, as shown in Fig. 9. Suppose that $V^{\prime}\left( x \right) = 0$; then,

7 $$ 4x^{3} + 2ux + v = 0 $$

**Figure 10:**
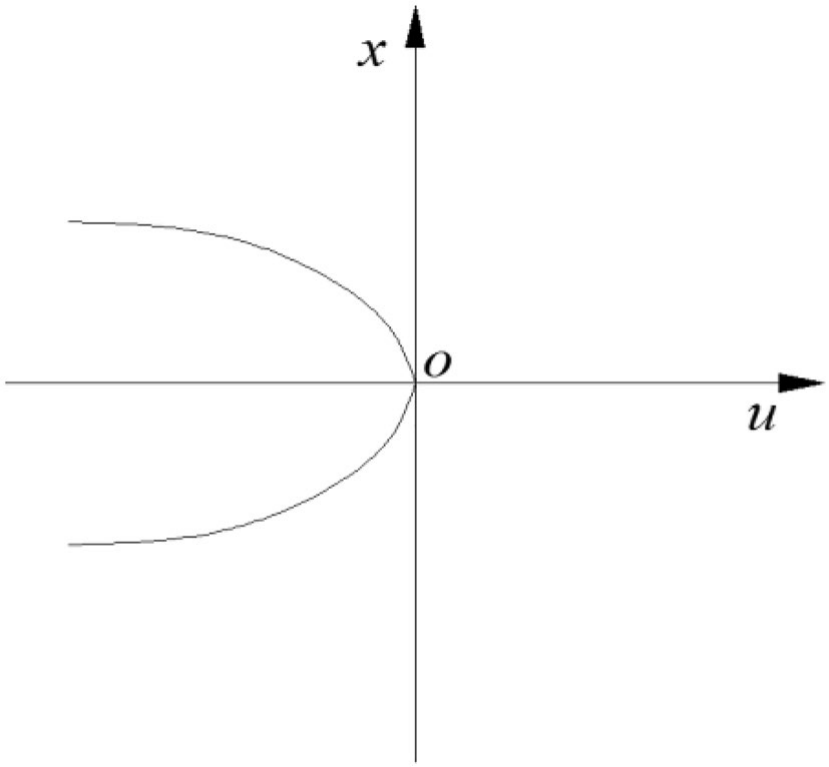
Variation of x with u in a singularity set.

According to Eq. (7), the equilibrium surface (M) can be obtained, and the set of folded or cusp inflection points of the equilibrium surface is called the set of singularities. From $V^{\prime\prime}\left( x \right) = 0$, as shown in Fig. 10, we have:

8 $$ 12x^{2} + 2u = 0 $$

The projection of the singularity set (S) on the O-UV plane is called the bifurcation set (B), which can be obtained from the discriminant of Eq. (7). Equation (7) is cubic, and its real root discriminant can be written as

9 $$ \Delta = 8u^{3} + 27v^{2} $$

According to Eq. (9), when $\Delta > 0$, x in Eq. (7) has a real root and a pair of conjugated virtual roots. When $\Delta = 0$, x in Eq. (7) has three real roots, two of which are equal. When $\Delta < 0$, x in Eq. (7) has three unequal real roots.

When $\Delta = 0$, the bifurcation set of the cusp mutation model is a half cubic parabola in the plane of control variables, and there is a cusp at (0,0) in the middle. According to Fig. 10, bifurcation set (B) divides the control variable plane into two regions, denoted as G and H. When control variables u and V are in region G, i.e., $\Delta > 0$, V is a stable equilibrium. When control variables U and V are in bifurcation set B, i.e., $\Delta = 0$, V is in the critical state of stable equilibrium and unstable equilibrium. When control variables u and V are in region H, i.e., $\Delta < 0$, since this region corresponds to the upper, middle and lower regions of the equilibrium surface, where the upper lobe and lower lobe are in stable equilibrium and the middle lobe is in unstable equilibrium, the condition of unstable equilibrium of V is $V^{\prime\prime}\left( x \right) < 0$.

### Mutation analysis of slope deformation

The slope deformation data are discrete data, and the slope deformation data are usually processed by accumulative smoothing as the initial data of the slope deformation mutation analysis^30^. Then, the cumulative sequence is expanded into a power series form by Taylor’s formula,

10 $$ x^{\left( 1 \right)} \left( t \right) = A_{0} + A_{1} t + A_{2} t^{2} + A_{3} t^{3} + \cdots + A_{n} t^{n} $$

After taking the first five terms of Eq. (10), we can conclude that

11 $$ x^{\left( 1 \right)} \left( t \right) = A_{0} + A_{1} t + A_{2} t^{2} + A_{3} t^{3} + A_{4} t^{4} + A_{5} t^{5} $$

According to Eq. (11), the change over time (t) will be

12 $$ \left\{ {\begin{array}{*{20}c} {x^{\left( 1 \right)} \left( {t_{1} } \right) = A_{0} + A_{1} t_{1} + A_{2} t_{1}^{2} + A_{3} t_{1}^{3} + A_{4} t_{1}^{4} + A_{5} t_{1}^{5} } \\ {x^{\left( 1 \right)} \left( {t_{2} } \right) = A_{0} + A_{1} t_{2} + A_{2} t_{2}^{2} + A_{3} t_{2}^{3} + A_{4} t_{2}^{4} + A_{5} t_{2}^{5} } \\ \vdots \\ {x^{\left( 1 \right)} \left( {t_{n} } \right) = A_{0} + A_{1} t_{n} + A_{2} t_{n}^{2} + A_{3} t_{n}^{3} + A_{4} t_{n}^{4} + A_{5} t_{n}^{5} } \\ \end{array} } \right. $$

Then, the matrix form of Eq. (12) is

13 $$ x^{\left( 1 \right)} = C\left( {\begin{array}{*{20}c} {A_{0} } & {A_{1} } & {A_{2} } & {A_{3} } & {A_{4} } & {A_{5} } \\ \end{array} } \right)^{T} $$

where $$C = \left[ {\begin{array}{*{20}c} 1 & {t_{1} } & {t_{1}^{2} } & {t_{1}^{3} } & {t_{1}^{4} } & {t_{1}^{5} } \\ 1 & {t_{2} } & {t_{2}^{2} } & {t_{2}^{3} } & {t_{2}^{4} } & {t_{2}^{5} } \\ \vdots & \vdots & \vdots & \vdots & \vdots & \vdots \\ 1 & {t_{n} } & {t_{n}^{2} } & {t_{n}^{3} } & {t_{n}^{4} } & {t_{n}^{5} } \\ \end{array} } \right]$$.

According to the least square method, the following equation can be obtained,

14 $$ \left( {\begin{array}{*{20}c} {A_{0} } & {A_{1} } & {A_{2} } & {A_{3} } & {A_{4} } & {A_{5} } \\ \end{array} } \right)^{T} = \left( {C^{T} C} \right)^{ - 1} C^{T} x^{\left( 1 \right)} $$

Based on cusp mutation theory, the accuracy of the model can be guaranteed by taking the first four terms of the Taylor series. Thus, the derivative of Eq. (11) can be obtained:

15 $$ x = x^{\left( 0 \right)} \left( t \right) = A_{1} + 2A_{2} t + 3A_{3} t^{2} + 4A_{4} t^{3} + 5A_{5} t^{4} $$

By assigning

16 $$ \left\{ {\begin{array}{*{20}c} {a_{0} = A_{{1}} } \\ {a_{{1}} = {2}A_{{2}} } \\ {a_{{2}} = {3}A_{{3}} } \\ {a_{{3}} = {4}A_{{4}} } \\ {a_{{4}} = {5}A_{{5}} } \\ \end{array} } \right. $$

Equation (15) can be rewritten as

17 $$ x = a_{0} + a_{1} t + a_{2} t^{2} + a_{3} t^{3} + a_{4} t^{4} $$

In addition, to eliminate the third term in Eq. (17), we can make,

18 $$ \left\{ {\begin{array}{l} {t = T_{x} - q} \\ {q = \frac{{a_{3} }}{{4a_{4} }}} \\ \end{array} } \right. $$

Substituting Eq. (18) into Eq. (17) yields the following:

19 $$ \begin{aligned} x & = a_{4} q^{4} - a_{3} q^{3} + a_{2} q^{2} - a_{1} q + a_{0} \\ & \quad + \left( { - 4a_{4} q^{3} + 3a_{3} q^{2} - 2a_{2} q + a_{1} } \right)T_{x} \\ & \quad + \left( {6a_{4} q^{2} - 3a_{3} q + a_{2} } \right)T_{x}^{2} \\ & \quad + a_{4} T_{x}^{4} \\ \end{aligned} $$

Equation (19) can be rewritten as:

20 $$ x = b_{0} + b_{1} T_{x} + b_{2} T_{x}^{2} + b_{4} T_{x}^{4} $$

We obtain:

21 $$ \left\{ {\begin{array}{l} {b_{0} = a_{4} q^{4} - a_{3} q^{3} + a_{2} q^{2} - a_{1} q + a_{0} } \\ {b_{1} = - 4a_{4} q^{3} + 3a_{3} q^{2} - 2a_{2} q + a_{1} \begin{array}{*{20}c} {} \\ \end{array} } \\ {b_{2} = 6a_{4} q^{2} - 3a_{3} q + a_{2} \begin{array}{*{20}c} {} & {\begin{array}{*{20}c} {} & {} & {} \\ \end{array} } \\ \end{array} } \\ {b_{4} = a_{4} \begin{array}{*{20}c} {} & {} & {} & {} & {} & {\begin{array}{*{20}c} {} & {} & {} \\ \end{array} } \\ \end{array} } \\ \end{array} } \right. $$

Variable substitution is made for Eq. (20), i.e.,

22 $$ T_{x} = \left\{ {\begin{array}{*{20}c} {\left( {\frac{1}{{b_{4} }}} \right)^{\frac{1}{4}} T,} & {b_{4} > 0} \\ {\left( { - \frac{1}{{b_{4} }}} \right)^{\frac{1}{4}} T,} & {b_{4} < 0} \\ \end{array} } \right. $$

23 $$ u_{t} = \left\{ {\begin{array}{*{20}c} {b_{2} \left( {\frac{1}{{b_{4} }}} \right)^{\frac{1}{2}} ,} & {b_{4} > 0} \\ { - b_{2} \left( { - \frac{1}{{b_{4} }}} \right)^{\frac{1}{2}} ,} & {b_{4} < 0} \\ \end{array} } \right. $$

24 $$ v_{t} = \left\{ {\begin{array}{*{20}c} {b_{1} \left( {\frac{1}{{b_{4} }}} \right)^{\frac{1}{4}} ,} & {b_{4} > 0} \\ { - b_{1} \left( { - \frac{1}{{b_{4} }}} \right)^{\frac{1}{4}} ,} & {b_{4} < 0} \\ \end{array} } \right. $$

Then, Eq. (20) can be written as

25 $$ x = \left\{ {\begin{array}{*{20}c} {T^{4} + u_{t} T^{2} + v_{t} T + b_{0} ,b_{4} > 0} \\ { - T^{4} - u_{t} T^{2} - v_{t} T + b_{0} ,b_{4} < 0} \\ \end{array} } \right. $$

where, T is the state variable (indirect time state variable), *b*_0_ is the parameter (which does not affect the analysis of mutation), and *u*_t_ and *v*_t_ are control variables. Meanwhile, from $\frac{{{\text{d}} x}}{{{\text{d}} T}} = 0$ and $\frac{{{\text{d}}^{2} x}}{{{\text{d}} T^{2} }} = 0$, the equation of the equilibrium surface and equation of the singularity set of Eq. (25) can be obtained, respectively,

26 $$ 4T^{3} + 2u_{t} T + v_{t} = 0 $$

27 $$ 12T^{2} + 2u_{t} = 0 $$

According to Eq. (26), the discriminant is,

28 $$ \Delta_{t} = 8u_{t}^{3} + 27v_{t}^{2} $$

Substituting Eqs. (16), (18) and (21) into Eqs. (23) and (24), and substituting the obtained results into Eq. (28) yield the following:

29 $$ u_{t} = \left\{ {\begin{array}{*{20}c} {\left( {3A_{3} - \frac{{6A_{4}^{2} }}{{5A_{5} }}} \right)\left( {\frac{1}{{5A_{5} }}} \right)^{\frac{1}{2}} ,} & {A_{5} > 0} \\ {\left( {\frac{{6A_{4}^{2} }}{{5A_{5} }} - 3A_{3} } \right)\left( { - \frac{1}{{5A_{5} }}} \right)^{\frac{1}{2}} ,} & {A_{5} < 0} \\ \end{array} } \right. $$

30 $$ v_{t} = \left\{ {\begin{array}{*{20}c} {\frac{{8A_{4}^{3} - 30A_{3} A_{4} A_{5} + 50A_{2} A_{5}^{2} }}{{25A_{5}^{2} }}\left( {\frac{1}{{5A_{5} }}} \right)^{\frac{1}{4}} ,} & {A_{5} > 0} \\ { - \frac{{8A_{4}^{3} - 30A_{3} A_{4} A_{5} + 50A_{2} A_{5}^{2} }}{{25A_{5}^{2} }}\left( { - \frac{1}{{5A_{5} }}} \right)^{\frac{1}{4}} ,} & {A_{5} < 0} \\ \end{array} } \right. $$

31 $$ \Delta_{t} = \left\{ {\begin{array}{ll} {8\left[ {\left( {3A_{3} - \frac{{6A_{4}^{2} }}{{5A_{5} }}} \right)\left( {\frac{1}{{5A_{5} }}} \right)^{\frac{1}{2}} } \right]^{3} + 27\left[ {\frac{{8A_{4}^{3} - 30A_{3} A_{4} A_{5} + 50A_{2} A_{5}^{2} }}{{25A_{5}^{2} }}\left( {\frac{1}{{5A_{5} }}} \right)^{\frac{1}{4}} } \right]^{2} ,} & {A_{5} > 0} \\ {8\left[ {\left( {\frac{{6A_{4}^{2} }}{{5A_{5} }} - 3A_{3} } \right)\left( { - \frac{1}{{5A_{5} }}} \right)^{\frac{1}{2}} } \right]^{3} + 27\left[ { - \frac{{8A_{4}^{3} - 30A_{3} A_{4} A_{5} + 50A_{2} A_{5}^{2} }}{{25A_{5}^{2} }}\left( { - \frac{1}{{5A_{5} }}} \right)^{\frac{1}{4}} } \right]^{2} ,} & {A_{5} < 0} \\ \end{array} } \right. $$

According to the cusp mutation theory, there are two possible mutations of the slope state:

1. Δt = 0.
2. Δt < 0, and $$12T^{2} + 2u_{t} < 0$$.

Under condition (1), $12T^{2} + 2u_{t} = 0$. When *u*_t_ = 0, *v*_t_ = 0, and Eq. (26) has triple zero roots, i.e., *T*_1_ = *T*_2_ = *T*_3_ = 0. When *u*_t_ < 0, Eq. (26) has three real roots, two of which are equal, i.e.,

32 $$ \left\{ {\begin{array}{l} {T_{1} = 2\left( { - \frac{{u_{t} }}{6}} \right)^{\frac{1}{2}} } \\ {T_{2} = T_{3} = - \left( { - \frac{{u_{t} }}{6}} \right)^{\frac{1}{2}} } \\ \end{array} } \right. $$

**Figure 11:**
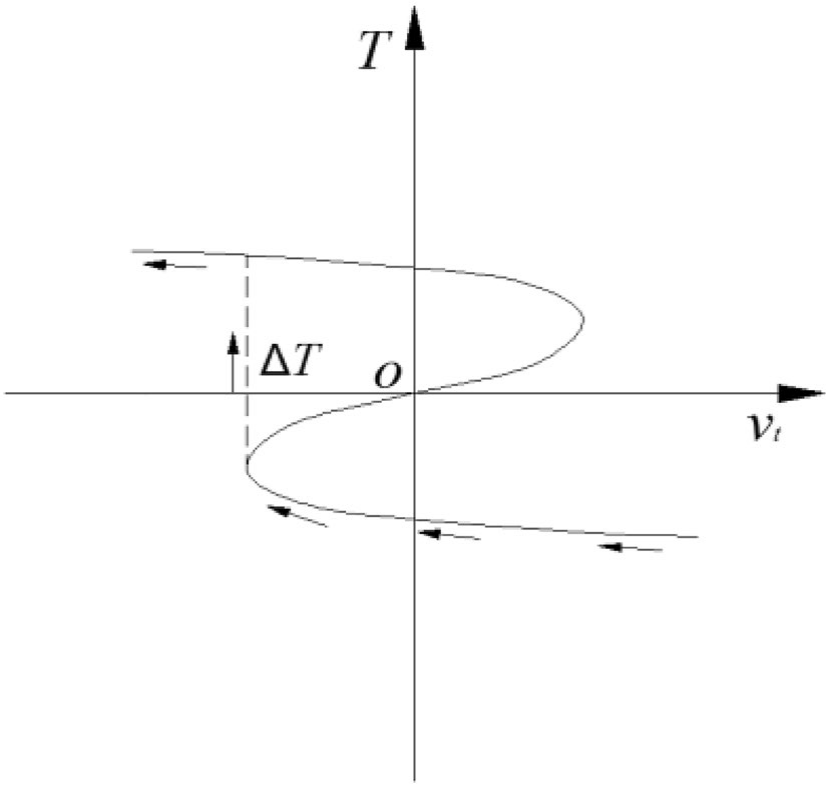
Jump form of state variable T.

When the control variable crosses the bifurcation set, the state variable T jumps, as shown in Fig. 11. The difference value of a sudden jump is

33 $$ \Delta T = 3\left( { - \frac{{u_{t} }}{6}} \right)^{\frac{1}{2}} $$

Thus, the time difference between the critical state of slope deformation and the abrupt instability state of slope deformation is

34 $$ \Delta t = \left\{ {\begin{array}{*{20}c} {\frac{{\sqrt {36A_{4}^{2} - 90A_{3} A_{5} } }}{{10A_{5} }},} & {A_{5} > 0} \\ {\frac{{\sqrt {36A_{4}^{2} - 90A_{3} A_{5} } }}{{ - 10A_{5} }},} & {A_{5} < 0} \\ \end{array} } \right. $$

Under condition (2), when Δ_t_ < 0 and $12T^{2} + 2u_{t} < 0$, Slope control variables are in the bifurcation set, and the actual time of mutation of slope deformation is generally less than the sum of Δ*t* and the duration of the critical state, i.e.,

35 $${t}_{mutation}<\left\{\begin{array}{c}\frac{2\sqrt{{36A}_{4}^{2}-{90A}_{3}{A}_{5}}-3{A}_{4}}{{15A}_{5}},{A}_{5}>0\\ \frac{2\sqrt{{36A}_{4}^{2}-{90A}_{3}{A}_{5}}+3{A}_{4}}{{-15A}_{5}},{A}_{5}<0\end{array}\right.$$

### Optimization and improvement of the cusp mutation model

According to the slope deformation cusp mutation model, whether the slope deformation state has mutation are directly related to the slope deformation data. However, the measured slope deformation data is very discrete, and it is difficult to eliminate it by one-time accumulation processing, which directly affects the establishment of the potential function of the cusp mutation model and causes the inaccurate prediction of the mutation model. In addition, the mutation model cannot determine the direction of slope mutation, i.e., it can only estimate whether the slope is unstable. Because the slope deformation sequence is based on the zero of deformation, and the zero of deformation is considered the stable point, the mutation point must be the unstable point. Therefore, it is unreasonable to determine whether the slope is unstable only by using the Δ value.

Based on these deficiencies, the virtual reality grey trend-cusp mutation model (short for virtual reality-mutation model) was proposed. Based on the idea of the relative change in the slope deformation trend, the relative potential function of the slope deformation virtual reality grey trend was established; then, the slope deformation mutation problem was solved.

The steps of establishing the slope deformation virtual reality-mutation model are as follows:

1. From the data of x(1)(i), slope deformation sequence $x^{\left( 1 \right)} = \left\{ {x^{\left( 1 \right)} \left( 1 \right),x^{\left( 1 \right)} \left( 2 \right), \ldots ,x^{\left( 1 \right)} \left( n \right)} \right\}$ shows an obvious trend change. Thus, divide the slope deformation sequence into two groups and (overlapping one data point to maintain the continuity of data) as follows:
  36 $$ x_{q}^{\left( 1 \right)} = \left\{ {x^{\left( 1 \right)} \left( 1 \right),x^{\left( 1 \right)} \left( 2 \right), \ldots ,x^{\left( 1 \right)} \left( {i - 1} \right)} \right\} $$
  37 $$ x_{h}^{\left( 1 \right)} = \left\{ {x^{\left( 1 \right)} \left( {i - 1} \right),x^{\left( 1 \right)} \left( i \right), \ldots ,x^{\left( 1 \right)} \left( n \right)} \right\} $$
2. Perform grey modelling for the two sequences and obtain the fitting and prediction sequences of the entire sequence. The fitting and prediction sequences of $x_{q}^{\left( 1 \right)}$ are
  38 $$ \left\{ {\begin{array}{*{20}c} {\hat{x}_{qn}^{\left( 1 \right)} = \left\{ {\hat{x}^{\left( 1 \right)} \left( 1 \right),\hat{x}^{\left( 1 \right)} \left( 2 \right), \ldots ,\hat{x}^{\left( 1 \right)} \left( {i - 1} \right)} \right\}} \\ {\tilde{x}_{qy}^{\left( 1 \right)} = \left\{ {\tilde{x}^{\left( 1 \right)} \left( i \right),\tilde{x}^{\left( 1 \right)} \left( {i + 1} \right), \ldots ,\tilde{x}^{\left( 1 \right)} \left( n \right)} \right\}} \\ \end{array} } \right. $$
  The fitting and prediction sequences of $x_{h}^{\left( 1 \right)}$ are
  39 $$ \left\{ {\begin{array}{*{20}c} {\tilde{x}_{hy}^{\left( 1 \right)} = \left\{ {\tilde{x}^{\left( 1 \right)} \left( 1 \right),\tilde{x}^{\left( 1 \right)} \left( 2 \right), \ldots ,\tilde{x}^{\left( 1 \right)} \left( {i - 1} \right)} \right\}} \\ {\hat{x}_{hn}^{\left( 1 \right)} = \left\{ {\hat{x}^{\left( 1 \right)} \left( i \right),\hat{x}^{\left( 1 \right)} \left( {i + 1} \right), \ldots ,\hat{x}^{\left( 1 \right)} \left( n \right)} \right\}} \\ \end{array} } \right. $$
  If a reality trend grey sequence is assigned as follows, $\hat{x}_{s}^{\left( 1 \right)} = \left\{ {\hat{x}_{qn}^{\left( 1 \right)} ,\hat{x}_{hn}^{\left( 1 \right)} } \right\}$, then
  40 $$ \hat{x}_{s}^{\left( 1 \right)} = \left\{ {\hat{x}^{\left( 1 \right)} \left( 1 \right),\hat{x}^{\left( 1 \right)} \left( 2 \right), \ldots ,\hat{x}^{\left( 1 \right)} \left( n \right)} \right\} $$
  If a virtual trend grey sequence is assigned as follows, $\tilde{x}_{x}^{\left( 1 \right)} = \left\{ {\tilde{x}_{qy}^{\left( 1 \right)} ,\tilde{x}_{hy}^{\left( 1 \right)} } \right\}$, then
  41 $$ \tilde{x}_{x}^{\left( 1 \right)} = \left\{ {\tilde{x}^{\left( 1 \right)} \left( 1 \right),\tilde{x}^{\left( 1 \right)} \left( 2 \right), \ldots ,\tilde{x}^{\left( 1 \right)} \left( n \right)} \right\} $$
  Thus, the difference value sequence of the virtual reality grey trend is,
  42 $$ x_{c}^{\left( 1 \right)} = \hat{x}_{s}^{\left( 1 \right)} - \tilde{x}_{x}^{\left( 1 \right)} = \left\{ {\hat{x}^{\left( 1 \right)} \left( 1 \right) - \tilde{x}^{\left( 1 \right)} \left( 1 \right),\hat{x}^{\left( 1 \right)} \left( 2 \right) - \tilde{x}^{\left( 1 \right)} \left( 2 \right), \ldots ,\hat{x}^{\left( 1 \right)} \left( n \right) - \tilde{x}^{\left( 1 \right)} \left( n \right)} \right\} $$
3. Select a group of difference value sequences with length 6 from the neighbourhood of the ith data of the sequence ($x_{c}^{\left( 1 \right)}$) for the Taylor power series expansion. Construct the relative potential function of the slope deformation virtual reality grey trend. Then, perform the slope deformation trend mutation analysis according to the cusp mutation model and calculate the critical time and mutation time before and after the trend change (because the calculation steps of the theoretical cusp mutation are identical to those mentioned above, they are not described here).

## Results

### Deformation mutation characteristics of the Damao slope based on mutation theory

**Figure 12:**
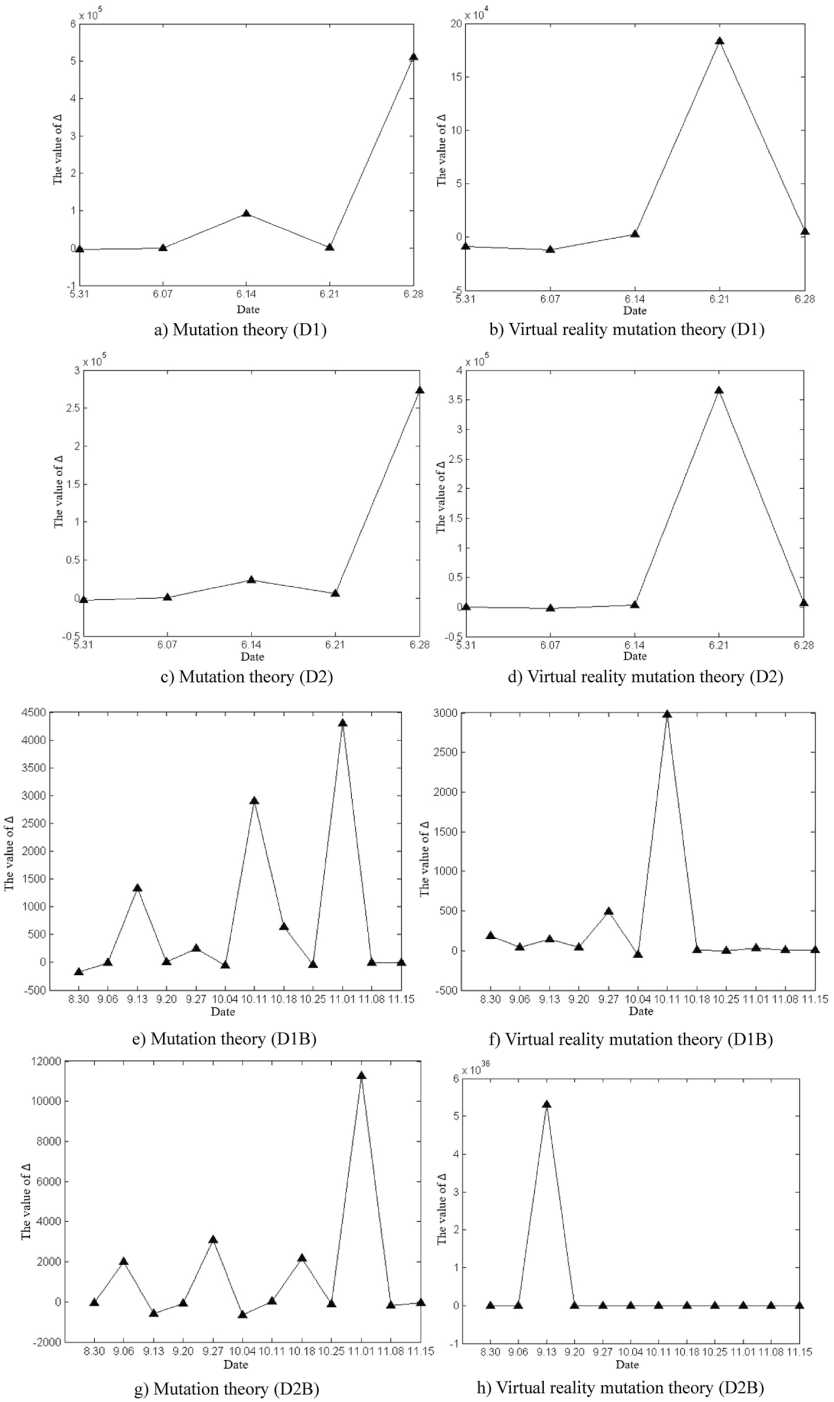

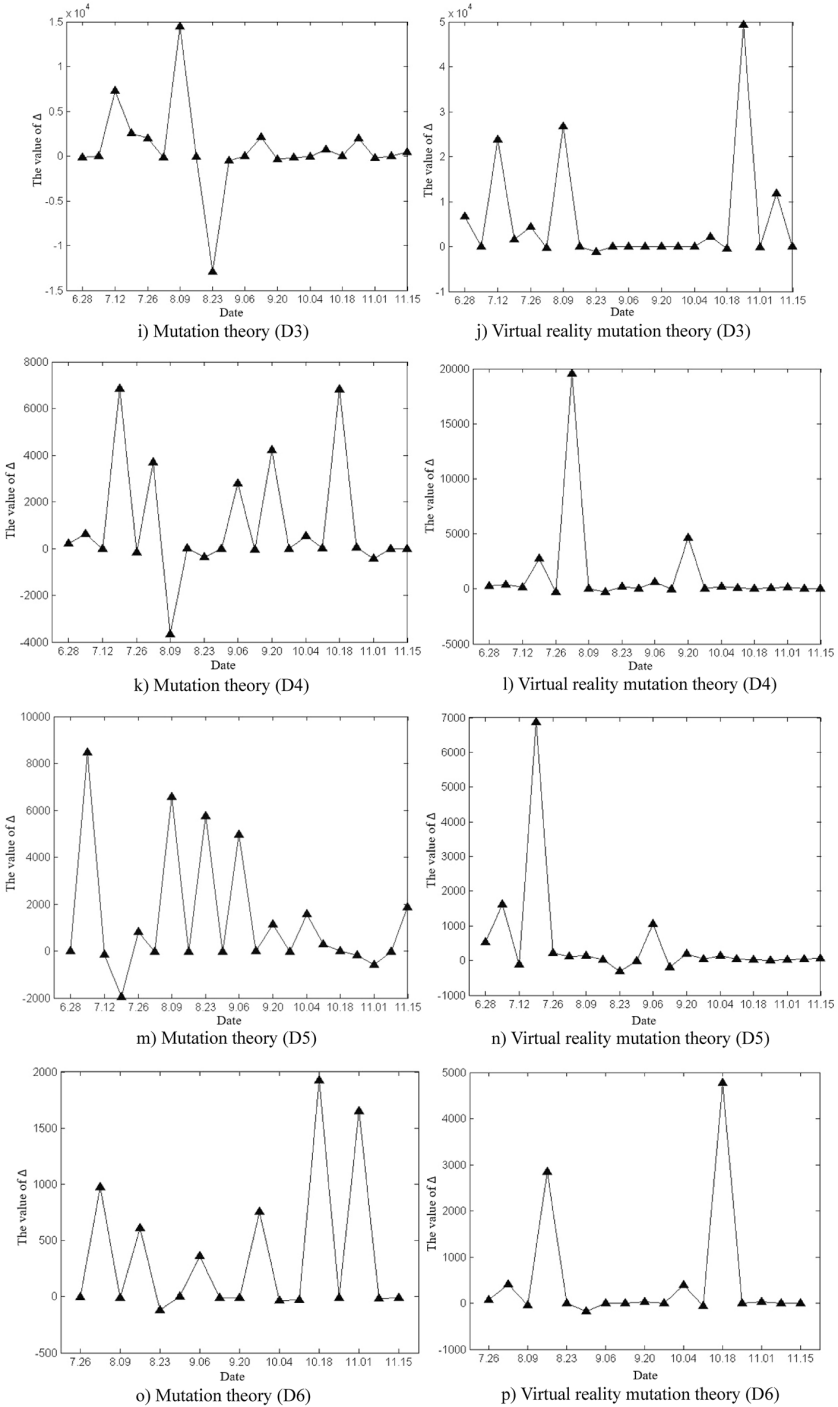

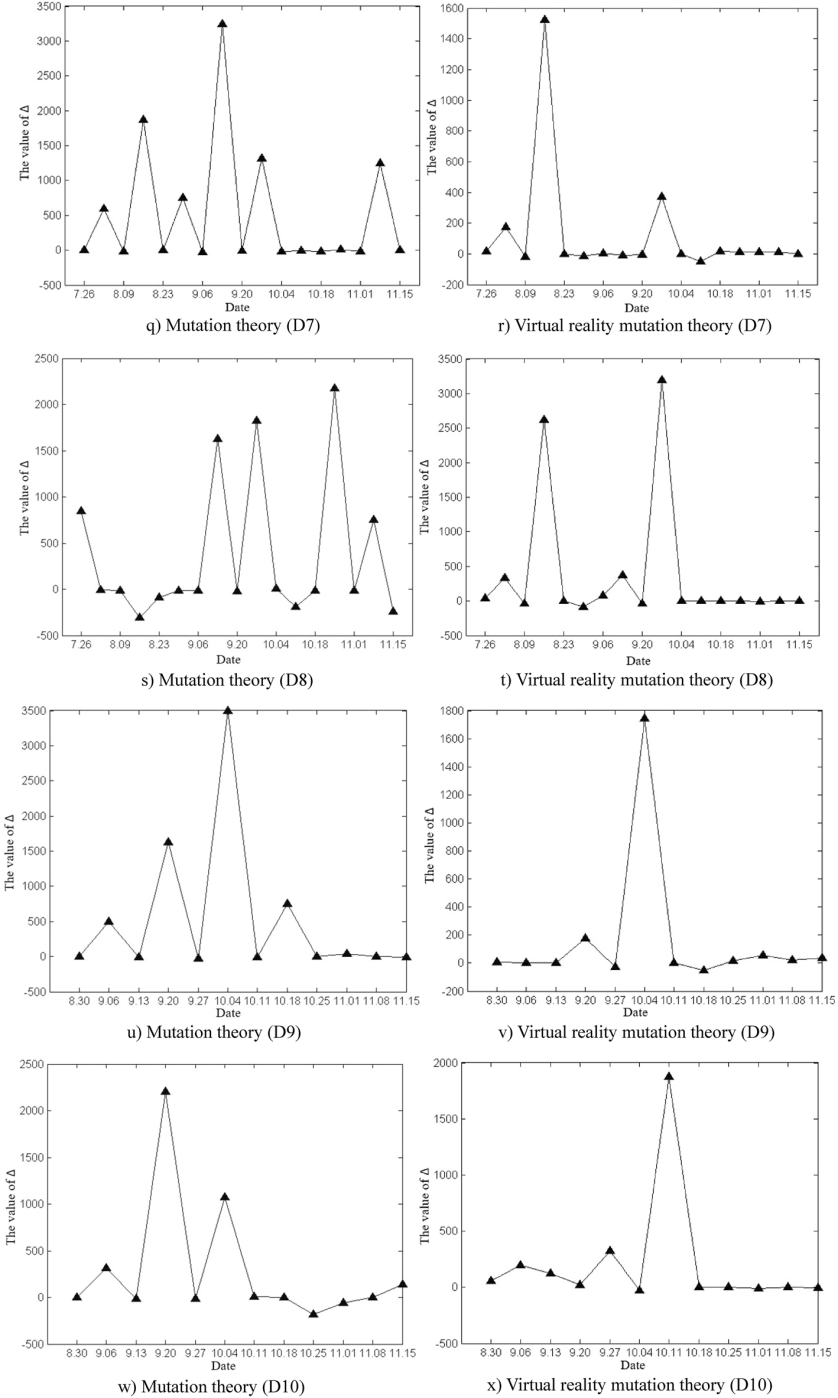

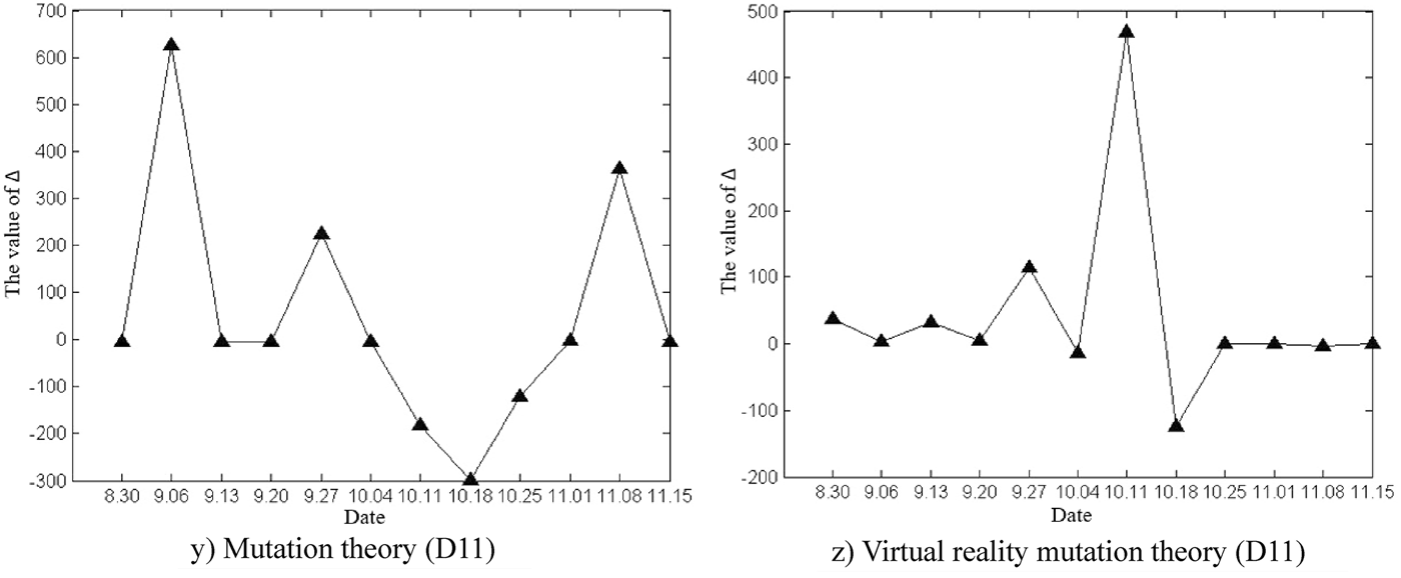
Time variation curve of the Δ value.

The deformation and mutation study of the Damao slope is mainly based on the original cusp mutation model and the proposed virtual reality mutation model in this paper. The variation characteristics of Δ values in slope deformation and mutation study by the two methods were analysed, as shown in Fig. 12. According to the calculated critical time and mutation time of slope deformation, the advantages and disadvantages of the two methods in the study of mutation warning of the deformation state of the Damao slope were comprehensively compared.

When the potential function model was established, the length of modelling data was set as 6. However, the data length of each monitoring point was greater than 6. Therefore, the method of data metabolic replacement was adopted to analyse the mutation of all monitoring points in the entire period.

As shown in Fig. 12, the time variation curve of Δ the value is calculated by mutation theory and virtual reality mutation theory. The time variation curve of Δ the value calculated by mutation theory greatly fluctuates, which indicates that the control variables u_t_ and v_t_ strongly change with time, which reflects the unstable characteristics of the Damao slope deformation. The time variation curve of the Δ value calculated by virtual reality mutation theory weakly fluctuates, which indicates that the virtual reality mutation theory can effectively stabilize the slope deformation data, filter out some interference, and make the Damao slope deformation mutation analysis more targeted.

According to mutation theory, when Δ < 0, slope instability failure will occur. In fact, near monitoring points D9, D10 and D11, the Δ values of slope deformation repeatedly appear to be negative, but the slope tends to be stable without an obvious instability phenomenon. The prediction based on the mutation model is obviously inconsistent with the actual situation.

This phenomenon can be explained by the virtual reality mutation model. When Δ < 0, the slope deformation trend or state abruptly changes. In other words, the slope changes from one type of deformation state or trend to another type of deformation state or trend, e.g., convergence–divergence mutation type (C–D), divergence–convergence mutation type (D–C), convergence–convergence mutation type (C–C), and divergence–divergence mutation type (D–D). Thus, the deformation and mutation forms of the Damao slope are mainly the C–D mutation type, D–C mutation type and C–C mutation type, as shown in Table 5. Among them, the C–D mutation type greatly affects the deformation state of the Damao slope and easily causes its cracking and failure.

**Table 5:** Comparison table of deformation and mutation time at each monitoring point.

Monitoring points	Inflection point of trend	Mutation theory		Virtual reality mutation theory					Situations in the field
		Critical time /w	Mutation time /w	Critical time /w	Mutation time /w	Before the mutation	Direction	After the mutation	
D1	4	< 5.06	< 11.04	< 4.44	< 8.61	Convergence	Negative	Divergency	The slope cracked from 8.4 weeks
D2	4	< 4.77	< 10.08	< 4.61	< 8.24				The slope cracked from 8.1 weeks
D1B	9	< 8.32	< 11.32	< 8.40	< 11.46		Positive	Convergence	No abnormalities
D2B	8	< 8.21	< 10.65	< 7.39	< 10.44				no abnormalities
D3	4	< 3.41	< 6.71	< 4.64	< 7.38		Negative	Divergency	The slope cracked from 6.4 weeks
	9	< 8.37	< 11.31	< 8.43	< 11.50	Divergency	Positive	Convergence	No abnormalities
	20	< 19.52	< 22.07	< 19.23	< 22.06	Convergence			
D4	8	< 7.60	< 10.78	< 7.42	< 10.50	Divergency			
	14	< 14.47	< 17.14	< 14.60	< 17.51	Convergence	Negative		
D5	6	< 5.76	< 9.03	< 7.42	< 10.50	Divergency	Positive		
	13	< 14.47	< 17.14	< 14.60	< 17.51	Convergence	Negative		
D6	6	< 6.35	< 9.50	< 6.40	< 9.44	Divergency	Positive		
	15	< 14.19	< 17.32	< 14.31	< 17.23	Convergence			
D7	6	< 5.36	< 8.25	< 5.35	< 8.33	Divergency			
	12	< 11.44	< 14.04	< 11.31	< 14.24	Convergence			
D8	6	< 5.62	< 8.97	< 5.38	< 8.40	Divergency			
	12	< 11.52	< 14.35	< 11.33	< 14.30	Convergence			
D9	8	< 7.33	< 10.08	< 7.36	< 10.36				
D10	9	< 7.46	< 10.33	< 8.39	< 11.44				
D11	9	< 8.09	< 11.72	< 8.45	< 11.59				

The critical time and mutation time of each monitoring point in the table are calculated by taking the starting time of each monitoring point as the initial zero point.

Table 5 shows that the difference between critical time and mutation time obtained by the virtual reality mutation theory and mutation theory is not obvious. Compared with mutation theory, the virtual reality mutation theory increases the analysis of the process of slope mutation deformation. Thus, the blindness of slope deformation mutation judgement is reduced. Therefore, in the early warning analysis of the deformation and mutation of the Damao slope, the virtual reality mutation theory is more consistent with the actual conditions of slope deformation and failure.

### Early warning of deformation and mutation of the Damao slope

In engineering, the warning control of slope deformation mainly includes deformation rate control and cumulative deformation control. In practical applications, slope deformation is generally controlled by combining the deformation rate with the accumulated deformation. In this paper, according to the empirical formula of the slope sliding limit deformation rate proposed by Broadbent^30^

43 $$ v_{c} = K^{2} v_{0} $$

where, v_C_ is the final slip velocity of the slope deformation failure section (divergent section); v_0_ is the initial deformation velocity of the slope deformation failure section (divergent section); K is a constant and if generally 4.6–10.4.

According to the deformation data at each monitoring point on the Damao slope, the initial velocities of monitoring points D1 and D2 at their failure sections are 5.6 mm/week and 6.4 mm/week, respectively. According to Eq. (43), when the slope slips, the final rate range of monitoring points D1 and D2 is 118.5–605.7 mm/week and 135.4–692.2 mm/week, respectively.

Based on the grey upper and lower limit segmentation prediction model, the failure sections of monitoring points D1 and D2 were predicted, and the deformation velocity prediction models at the failure sections of monitoring points D1 and D2 were obtained as follows:

44 $$ v_{{{\text{D1}}}} = 6.6170 \cdot e^{0.4511t} $$

45 $$ v_{{{\text{D2}}}} = 5.6447 \cdot e^{0.5437t} $$

Let v_D1_ and v_D2_ be equal to v_c_; then, the total time of slope sliding at monitoring points D1 and D2 is

46 $${t}_{totalD1}={t}_{destructionD1}+4$$

47 $${t}_{totalD2}={t}_{destructionD2}+4$$

48 $${t}_{destructionD1}=2.2168\cdot ln\frac{{K}^{2}{v}_{oD1}}{6.6170}$$

49 $${t}_{destructionD2}=1.8392\cdot ln\frac{{K}^{2}{v}_{oD2}}{5.6447}$$

Thus, the sliding time of the Damao slope at monitoring point D1 is 10.4–14.0 weeks, which is 2.0–5.6 weeks later than the cracking time. The sliding time of the Damao slope at monitoring point D2 is 9.8–12.8 weeks, which is 1.7–4.7 weeks later than its cracking time. Therefore, the process of catastrophic instability of the Damao slope can be inferred, which mainly includes slope excavation disturbance, slope deformation start, slope cracking failure, slope sliding and instability.

The cumulative deformation prediction formulas of monitoring points D1 and D2 at the failure sections were obtained based on the grey upper and lower limit segmentation prediction models respectively

50 $$ x_{{{\text{D}} 1}} = 14.6685 \cdot e^{0.4511t} - 2.7835 $$

51 $$ x_{{{\text{D}} 2}} = 10.3821 \cdot e^{0.5437t} + 4.5832 $$

By substituting Eqs. (48) and (49) into Eqs. (50) and (51) respectively, we observe that the accumulated deformation of slope sliding at monitoring point D1 is 260.4–1332.2 mm, and that of slope sliding failure at monitoring point D2 is 247.7–1246.8 mm.

Combined with the deformation characteristics of the Damao slope itself, the lower limit of the average deformation velocity and average cumulative deformation of monitoring points D1 and D2 at sliding failure were taken as the critical control warning values of its catastrophic deformation, i.e., the critical deformation velocity of the Damao slope is 127.0 mm/week, and the critical cumulative deformation is 254.1 mm.

## Conclusion and discussion

1. Based on the principle of error analysis, the deformation monitoring points of karst slope are arranged in areas with thick karst overburden, dissolution and fragmentation, cave development, slope cracking and faults or weak interlayers. In addition, according to the karst geological characteristics of the Damao slope, the deformation monitoring points adopt the layout form of “dense at the top and sparse at the bottom”.
2. Mutation analysis of slope deformation data is performed by the mutation model. Because the mutation model is often inconsistent with the actual slope deformation, an improved virtual reality mutation model is proposed, considering that mutation can be converted between two deformation trends or states, such as convergence–divergency mutation type (C–D), divergence–convergence mutation type (D–C), convergence–convergence mutation type (C–C), divergence–divergency mutation type (D–D). The application in the deformation and mutation of the Damao slope, shows that the deformation and mutation forms of the Damao slope are mainly the C–D mutation type, D–C mutation type and C–C mutation type. Among them, the C–D mutation type greatly affects the deformation state of the Damao slope and easily causes the cracking and failure of the Damao slope.
3. Based on the empirical formula of slope sliding limit deformation velocity and combined with the grey prediction model, the sliding time of the Damao slope at monitoring point D1 is 10.4–14.0 weeks, which is 2.0–5.6 weeks later than the cracking time. The sliding time of the Damao slope at monitoring point D2 is 9.8–12.8 weeks, which is 1.7–4.7 weeks later than its cracking time. In addition, the lower limit of the average deformation rate of 127.0 mm/week and the lower limit of the average cumulative deformation of 254.1 mm were taken as the critical control warning values of the catastrophic deformation of monitoring points D1 and D2. It provides a basis to quantitatively study the deformation index in similar engineering risk assessments.

## Data Availability

The data that support the findings of this study are available from Beijing Jiaotong University, but restrictions apply to the availability of these data, which were used under licence for the current study and are not publicly available. However, the data are available from the authors upon reasonable request and with permission of Beijing Jiaotong University.
